# Case of Acute Retinitis, Caused by the Use of the Microscope

**Published:** 1844-08-24

**Authors:** William White Cooper

**Affiliations:** Surgeon to the North London Ophthalmic Institution, &c.


*Case of Acute Retinitis, caused by the use of the Microscope.* By WILLIAM WHITE COOPER, ESQ. Surgeon to the North London Ophthalmic Institution, &c.

The following case is considered interesting by the author, as offering a warning to those microscopical investigators who are in the habit of pursuing their researches with powerful instruments, and the aid of strong and concentrated light. Mr. G——, a gentleman well known for his remarkable skill in minute and microscopical dissection, was engaged on Friday, the 29th of March last, dissecting the nerves of the human tongue, under a powerful microscope, and in a situation exposed to the full influence of the sun, which, although occasionally obscured, burst forth at times with great power. The nerves, having been cleanly dissected, were of a dazzling white, and, whilst he was intently regarding them through the microscope, the sun, which had previously been obscured, suddenly shone forth with all its brilliancy upon them. Acute pain was instantly felt in the eye, pervading the whole globe, so severe as to induce Mr. G. to start back and utter an exclamation. He paused from work, but for some time was not able to see any thing with that eye, the spectrum of the sun continuing before it whether closed or open. In about twenty minutes, however, this, as well as the pain, had sufficiently subsided to enable him to resume the work with the other eye; but the injured organ was not free from uneasiness until the evening. The following day the eye was not painful, and he incautiously used it to complete his dissection, when the very same occurrence took place as on the previous day—the reflected rays of the sun being thrown powerfully on the retina. This time the shock was excessive; great and deeply-seated pain pervading the whole globe, with much intolerance of light, immediately set in, and the spectrum of the sun was most distressing. He remained in acute suffering all the evening and the following night; and, on the next day (Sunday), it continued to increase, with a sensation of fulness and tenderness of the globe, and extreme intolerance of light. Fomentations failed to afford relief, and when he consulted Mr. Cooper on Monday morning, the following were the symptoms:—Acute, deep-seated pain in the eye; exquisite tenderness, especially at the upper half of the globe; great intolerance of light; profuse lachrymation; any attempt at vision produced luminous spectra; pupil contracted; iris natural; conjunctiva, but slightly injected; pulse, feeble and irritable: he complained of weakness and mental depression. He was sent to bed in a darkened room, and ordered to apply twelve leeches around the eye, to foment and take a purgative pill and dose. Mercury, he stated, always disagreed with him; and this, which is so important an auxiliary in such cases, was, therefore, obliged to be used with great caution. The next day he was rather easier; friction of the brow and temple, with mercurial ointment and opium, was directed. Pil. hydrarg. with conium, ordered at night, with salines and antimony at intervals. The following day all the symptoms were alleviated. The antimony was omitted, but the mercurials directed to be continued. On Thursday, a still greater improvement was manifested, the eye being perfectly free from pain, except when exposed to the light. There was, however, great debility and general exhaustion, and half a grain of quinine, twice a-day, with a moderate meat diet, was ordered; the mercurial friction being continued. This treatment, with counter-irritation behind the ear, and the use of a mild astringent collyrium, was steadily pursued for a week with advantage, although the least exertion of the eye immediately produced luminous spectra. The further treatment of the case presented nothing remarkable. The eye gradually and steadily recovered, and is now perfectly well.—From the state of general debility in which the system was at the time of the attack, taken in connection with the constitutional antipathy to mercury, the free exhibition of the medicine was inadmissible,—whereas the patient did perfectly well under its cautious use, the system being at the same time supported by a nutritious diet, and the careful exhibition of tonics.—*London Med. Times.*

